# Supplementary material for: Optic nerve sheath diameter in patients with hepatic encephalopathy
Source: PLoS One. 2022 Nov 16;17(11):e0277643. doi: 10.1371/journal.pone.0277643 (PMC9668157; doi:10.1371/journal.pone.0277643)
Supplement: Attachment — Submitted filename: response the reviewers.docx [file pone.0277643.s002.docx]

**Dear Editor;**

**We thank the Referees for their interest in our work and for their helpful comments on the manuscript and we have tried to do our best to respond to the raised points. We considered all comments and revised our manuscript according to your recommendations. All the changes marked as red on the marked copy. We explained how we changed the paper by answering the reviewer.**

**Best regards,**

**On behalf of all of the co-authors,**

**Neşe Çolak, MD, Assos.Prof.Dr.**

**School of Medicine, Department of Emergency Medicine, Dokuz Eylul University Medical Faculty, Izmir, Turkey**

**Address: Dokuz Eylul University School of Medicine, Department of Emergency Medicine, Balcova, 35340 Izmir, Turkey**

**Phone: +90 5055251110**

Reviewer's Comments and Answers

Reviewer #1:

- It is not appropriate to ask a question in the title of the article, change it

A: We revised the title of the manuscript as “Optic nerve sheath diameter in patients with hepatic encephalopathy” and Running title as a “Optic nerve sheath diameter in hepatic encephalopathy”

- In the Abstract Conclusion section, the authors said;"We have shown that ONSD increases in HE patients compared to the control group. and Our study demonstrates an association between increased ONSD in HE patients compared to the control group.'' Two sentences with the same meaning are written, please correct it.

**A: We revised the abstract and conclusion section as “We have shown that ONSD increases in HE patients compared to the control group. ONSD was not related to the Child-Pugh Score, HE grade, and in-hospital mortality”**

- Specify West Haven HE grade and Child-Pugh classification in detail in the material method section. In addition, specify how many points you get from each criterion in these classifications in detail in the results section.

**A: We added West Haven HE grade and Child-Pugh classification in detail in the material method section as**

**“The severity of HE was graded with the West Haven Criteria and Child-Pugh classification.  West Haven criteria categorize HE from grade 1 to grade 4. Grade 1: Mild lack of awareness, some subtle personality changes, and shortened attention span, Grade 2: Lethargy, disoriented, inappropriate behavior, obvious asterisks and slurred speech, Grade 3: Sleepy but somnolent but arousable, gross disorientation, inappropriate behavior, muscle rigidity, clonus and hyperreflexia, Grade 4: Coma and cerebral posture.**

**In the Child-Pugh classification, the degree of ascites (None = 1 point, slight = 2 points, moderate = 3 points), bilirubin level (< 2 mg/ml = 1 point, 2 to 3 mg/ml = 2 points, > 3 mg/ml = 3 points), albümin level (> 3.5mg/ml = 1 point, 2.8 to 3.5mg/ml = 2 points, < 2.8mg/ml = 3 points) and the prothrombin time (< 4 sec = 1 point, 4 to 6 sec = 2 points, > 6 sec = 3 points), and the degree of encephalopathy (None = 1 point, Grade 1 and 2 = 2 points, Grade 3 and 4 = 3 points) were evaluated. A child-Pugh score of 5 to 6 is was considered as class A, 7 to 9 is class B, and 10 to 15 is class C.****”**

- Show on the figure the image of a real patient that you have measured the optic nerve diameter on CT.

**A: Figure 1 added method section.**

-The authors stated that the in-hospital mortality rate was 24%. This rate seems too exaggerated to me. Is this rate for all patients diagnosed with Hepatic encephalopathy at the hospital or is it the rate of patients included in the study?

**A: This mortality rate (24%) belongs the patients included our study.**

Reviewer #2: The manuscript is well written, in a very detailed and clear manner. The methods are explained clearly and the flow is good overall. The conclusion is consistent with the evidence presented. The study sample size is small but it suggested interesting correlation between hepatic encephalopathy and optic nerve sheath diameter. More studies need to be done in this regard, to determine its prognostic value in severe hepatic encephalopathy cases. I strongly recommend publication of this manuscript.

**A: Thank you for your attention and appreciation.**

Reviewer #3: Your study is one of the first one done in adult population with liver cirrhosis and HE measuring ONSD with CT scan. I agree with your comment that your study may not have enough power to see any difference between different groups of Child Pugh Score or HE grade. It did not show any correlation in mortality outcome in liver cirrhosis patients but do you have any information on other parameters like length of stay which can be higher in more serious and complicated patients.

**A: Thank you for your attention. In our study, we focused on OSND and mortality rate/clinical severity scores, therefore we don’t evaluate other parameters such as length of hospital stay.**

Reviewer #4: Decent article but the Etio pathogenesis should have been more clearly explained. Explanation of etio pathogenesis is very short , it needs to be further elaborated for a clear understanding of why ONSD increases in HE

**A: We added about the etiopathogenesis in more detail and references in the introduction section as “There are several important hypotheses regarding the increase in intracranial pressure in HE. The most accepted hypothesis is that ammonia detoxifies glutamine in astrocytes and glutamine causes swelling and brain edema development in astrocytes by osmotic effect. Cytotoxicity in astrocytes causes disruption of the blood-brain barrier. The other hypothesis is that cerebral edema develops secondary to cerebral vasodilation in HE patients [12-13].”**
